# Persisting benefits 12–18 months after discontinuation of pubertal metformin therapy in low birthweight girls

**DOI:** 10.1111/j.1365-2265.2007.02952.x

**Published:** 2007-09

**Authors:** Ken Ong, Francis de Zegher, Carme Valls, David B Dunger, Lourdes Ibáñez

**Affiliations:** * Medical Research Council Epidemiology Unit Cambridge, UK; † Department of Paediatrics, University of Cambridge Cambridge, UK; ‡ Endocrinology Unit, Hospital Sant Joan de Déu, University of Barcelona Esplugues, Barcelona, Spain; § Hormonal Laboratory, Hospital Sant Joan de Déu, University of Barcelona Esplugues, Barcelona, Spain; ¶ Department of Pediatrics, University of Leuven Leuven, Belgium

## Abstract

**Background:**

Discontinuation of metformin therapy, if started beyond menarche in adolescents or young women with hyperinsulinaemia following low birthweight, is rapidly followed by rebound deteriorations in body fat, insulin resistance and blood lipid profile.

**Objective:**

We hypothesized that early commencement of metformin and its continuation throughout puberty might have more persisting benefits.

**Patients and measurements:**

We followed up on a previously reported randomized study cohort at 12 months and 18 months after treatment discontinuation, including body composition by absorptiometry, fasting insulin, glucose and blood lipids. In that open-labelled, prospective study, 22 low birthweight girls with early normal puberty (Stage 2 breast development at age 8–9 years) were randomized to remain untreated (*N* = 12]) or to receive metformin (850 mg/day; *N* = 10) for 36 months (between time –36 months to 0 month).

**Results:**

The significant improvements previously reported at the end of the 36-month active treatment period in per cent body fat, abdominal fat mass, fasting insulin sensitivity, high density lipoprotein (HDL) cholesterol and triglyceride levels all persisted at follow-up 12 months after treatment discontinuation. Further anthropometry at 18 months off therapy confirmed the persistence of benefits in height, body mass index (BMI) and waist circumference in the previously metformin-exposed girls.

**Conclusion:**

In low birth weight girls with early normal onset of puberty, metformin treatment for 3 years across puberty resulted in auxological, endocrine and metabolic benefits that persisted for at least 1 year after metformin withdrawal. Further follow-up and longer-term studies are needed to explore the possibility that insulin sensitization therapy during puberty might reprogramme predisposition to metabolic disease.

## Introduction

We recently reported that long-term insulin sensitization therapy with metformin during puberty in girls has cumulative auxological, body composition and endocrine-metabolic benefits.^1^

In that open-label randomized study, 22 girls aged 9 years with history of low birthweight and early normal onset of puberty received either metformin 850 mg (Dianben®, Merck, Farma y Química, Barcelona, Spain) once daily or remained untreated for 36 months. Metformin was associated with a leaner body composition and more favourable lipid profile.^1^ Strikingly, metformin prolonged the duration of puberty and also pubertal growth, and consequently adult height was increased by 4 cm^1^ Those results suggested that hyperinsulinaemia could underpin the rapid transit through puberty and loss of adult stature seen in low birthweight girls with early normal puberty.^2^

We now report on the follow-up of that randomized study cohort^1^ at 12 month and 18 month after discontinuation of the trial medication.

### Research design and methods

We report on the follow-up post-treatment discontinuation of a previously reported open-label study of girls from Barcelona, Spain, who were randomized to remain untreated (*N* = 12) or to receive metformin (850 mg once daily; *N* = 10) for 36 months.^1^ There were no significant pretreatment differences between Met(+) and Met(–) girls in any variable.^1^ Auxology, body composition by absorptiometry and fasting endocrine-metabolic markers were assessed at 12 months, and a further auxology-only assessment was performed at 18 months after treatment discontinuation, using the same protocols as previously described.^1^

The original inclusion criteria included: (1) birthweight below –1·5 standard deviation score (SDS); (2) early normal onset of breast development between age 8–9 years; (3) central puberty, by GnRH agonist test and pelvic ultrasound examination; and (4) height = 1 SD above mid-parental height SD. Exclusion criteria included familial or personal history of diabetes; evidence for precocious pubarche, thyroid dysfunction or glucose intolerance; and taking any medication known to affect gonadal function or carbohydrate metabolism.^1^ All girls remained off metformin during the 18-month follow-up period.

The study was registered as ISRCTN06805028 and was approved by the Institutional Review Board of St Joan University Hospital. Informed consent was obtained from the parents and assent from the minors.

Changes in all parameters from baseline were compared between the two groups by Mann–Whitney *U*-tests.

## Results

**Fig. 1:**
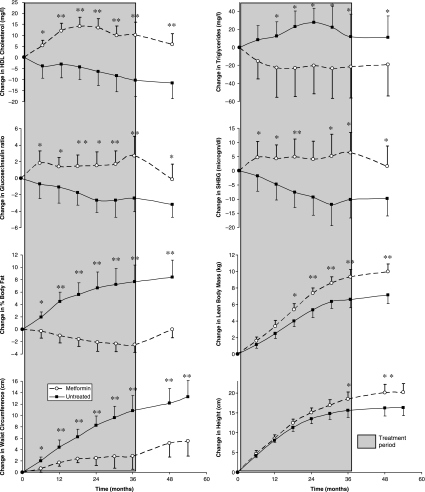
Changes in hormone-metabolic, body composition and anthropometric variables from pretreatment baseline (0 month), during the 36-month metformin 850 mg daily treatment study (shaded area; previously reported in^1^), and at 12 months and 18 months after treatment discontinuation (times: 48 months and 54 months). Means and 95% CI are shown. *P < 0·05 and **P < 0·005 by Mann–Whitney *U*-tests for the Untreated *vs.* Metformin groups at each time-point.

All the previously reported improvements in endocrine-metabolic, body composition and anthopometric variables apparent at the end of the 36-month active treatment period^1^ remained significantly different between the two groups at the 48 months assessment (i.e. 12 months after treatment discontinuation) (Fig. 1, Table 1).

**Table 1:** Clinical, endocrine-metabolic and body composition indices in girls with low birthweight and early normal pubertal onset, who were randomized to remain untreated (*N* = 12) or to receive metformin 850 mg daily (*N* = 10) for 36 months, and then followed up for 18 months after discontinuation of treatment

	0 month (treatment start)		36 months (treatment stop)		48 months (12 month post-treatment)			54 months (18 months post-treatment)	
					
	Untreated	Metformin	Untreated	Metformin	Untreated	Metformin	†Comparison of changes from 36–48 months	Untreated	Metformin
Chronological age (years)	9·1 ± 0·1	9·0 ± 0·1	12·2 ± 0·1	12·1 ± 0·1	13·2 ± 0·1	13·1 ± 0·1	NS	13·7 ± 0·1	13·6 ± 0·1
Mid-parental height (cm)	157·6 ± 1·3	159·6 ± 1·3	–	–	–	–	–	–	–
Height above MPH (cm)	–19·3 ± 1·2	–19·1 ± 1·6	–3·3 ± 0·9	0·4 ± 0·7***	–3·0 ± 1·0	0·9 ± 0·7***	0·02	–3·0 ± 1·0	1·1 ± 0·7***
BMI (kg/m^2^)	20·2 ± 0·8	21·0 ± 0·8	22·7 ± 0·5	21·6 ± 0·6***	23·2 ± 0·6	22·3 ± 0·5**	NS	23·6 ± 0·5	22·7 ± 0·5**
Waist circ. (cm)	64·3 ± 1·8	68·3 ± 2·3	75·1 ± 1·2	71·2 ± 1·5***	76·4 ± 1·2	73·4 ± 1·5***	0·02	77·5 ± 1·5	73·8 ± 1·4***
Fasting glucose (mg/dl)	84·7 ± 1·8	83·6 ± 1·8	93·7 ± 2·4	89·3 ± 1·0	91·2 ± 2·2	91·3 ± 1·3	0·004		
Fasting insulin (mU/l)	11·8 ± 1·6	14·2 ± 2·2	15·0 ± 1·7	10·1 ± 1·0*	16·2 ± 1·5	14·4 ± 1·4	0·01	–	–
Testosterone (ng/dl)	38·9 ± 4·0	30·2 ± 2·6	44·4 ± 4·7	38·1 ± 3·5	51·0 ± 3·1	45·2 ± 3·2	NS	–	–
SHBG (µg/dl)	1·2 ± 0·1	1·0 ± 0·1	0·9 ± 0·1	1·2 ± 0·1***	0·9 ± 0·1	1·1 ± 0·1**	0·0004	–	–
IGF-I (ng/ml)	298 ± 17	292 ± 23	268 ± 26	197 ± 6*	277 ± 22	231 ± 10	NS	–	–
HDL cholesterol (mg/l)	56 ± 4	51 ± 5	46 ± 2	61 ± 2***	44 ± 2	56 ± 2***	NS	–	–
LDL cholesterol (mg/l)	102 ± 5	93 ± 4	110 ± 4	90 ± 2**	116 ± 4	99 ± 3**	NS	–	–
Triglycerides (mg/l)	76 ± 11	80 ± 18	87 ± 6	58 ± 3***	87 ± 5	61 ± 3***	NS	–	–
Percent body fat (%)	31·5	34·2	39·1	31·7***	39·9	34·2***	0·00001	–	–
Abdominal fat mass (kg)	3·7 ± 0·5	4·7 ± 0·6	6·5 ± 0·5	4·9 ± 0·5***	7·0 ± 0·5	5·8 ± 0·6***	0·02	–	–
Lean body mass (kg)	25·2 ± 1·2	26·6 ± 1·0	31·8 ± 1·1	36·0 ± 0·8***	32·3 ± 1·1	36·6 ± 0·8***	NS	–	–
Bone mineral density (g/cm^2^)	0·78 ± 0·02	0·83 ± 0·03	1·06 ± 0·03	1·12 ± 0·02	1·11 ± 0·02	1·16 ± 0·02	NS	–	–

Values are mean ± SEM. MPH, mid-parental height; BMI, body mass index; SHBG, sex hormone binding globulin; IGF-I, insulin-like growth factor-I; HDL, high density lipoprotein; LDL, low density lipoprotein.

* *P* < 0·05

** *P* < 0·01

*** *P* < 0·001 for the Untreated *vs.* Metformin groups at each time-point. Comparisons at 36 months, 48 months and 54 months are adjusted for baseline pretreatment (0 month) values, using analysis of covariance. All comparisons at baseline pretreatment *P* > 0·1. NS, nonsignificant.

† Changes in each variable between 36 months and 48 months (the first 12 months post-treatment) were compared between Metformin and Untreated groups by using *t*-tests.

Not surprisingly, following discontinuation of insulin sensitization therapy there was an initial partial convergence between the two groups in levels of insulin resistance and sex hormone binding globulin (SHBG) from 36 months to 48 months. However, the size of benefit in HDL cholesterol and triglycerides levels remained unchanged during this 12-month period off treatment (Fig 1 and Table 1). Similarly, while there was some significant increase in percentage body fat during the 12 months following discontinuation of metformin (from 31·7% at 36 months to 34·2% at 48 months), the reduction compared to the untreated group remained sizable at 48 months (8·3% less gain in percentage body fat from baseline; Fig. 1).

At 54 months (18 months after discontinuation), a further assessment was made of anthropometry only. No further gains in height were seen in either group and the significant benefit in waist circumference persisted without any obvious further convergence between the two groups (Fig. 1, Table 1).

## Discussion

In low birthweight girls with early normal onset of puberty, metformin treatment for 36 months across puberty resulted in auxological, body composition, endocrine and metabolic benefits that persisted for at least 1 year after metformin discontinuation. The previously reported active treatment phase of this study tested the hypothesis that pubertal insulin sensitization delays menarche and prolongs height gains in these girls.^1^ That study also demonstrated the role of insulin on the pubertal changes in body composition and blood lipid profile.^1^

The current observations are in contrast to previous studies in older postmenarcheal girls aged 12–18 years and in young women with history of low birthweight and precocious pubarche in whom reversal of the body composition and endocrine-metabolic benefits of metformin, with or without flutamide, were rapidly apparent within 3–6 months off treatment.^3^–^5^ Those studies suggested that continuous metformin therapy was necessary in such low birthweight girls.^3^–^5^ Our present findings therefore raise the possibility that early introduction of insulin sensitization therapy and its continuation throughout puberty might have long-term benefits, or even possibly reprogramme^6^ the predisposition towards adverse metabolic disease risk in these low birthweight girls.

An alternative explanation might be that previous metformin therapy in these girls delayed not only their pubertal progression, but also simply delayed their age-related increase in hyperinsulinaemia. At the 48-month follow-up, metformin-exposed girls were on average 2 years beyond menarche compared to 3 years postmenarche in the untreated girls, and it is possible that they have an overall slower tempo of growth, maturation and also metabolic deterioration. Reassuringly, between 12 months and 18 months the metformin-exposed girls maintained their lower BMI and waist circumference compared to previously untreated girls. However, further repeated endocrine-metabolic assessments and other longer-term studies will be needed to demonstrate the duration of benefit of pubertal metformin therapy.

Furthermore, the study was unblinded and included a small number of patients. However, with the current knowledge at the time of recruitment we considered that it would have been difficult to perform a blinded placebo-controlled study over such a long timescale in children, and although dietary records were not obtained at any time-point, there were no obvious differences in lifestyle or behaviour observed. Further studies will also need to investigate whether our findings in girls with history of low birthweight and early puberty are representative of other childhood populations characterized by insulin resistance, such as children with low birthweight, postnatal catch-up growth, and/or obesity.^7^, ^8^

In conclusion, in low birthweight girls with early normal puberty, pubertal metformin treatment appears to have persisting endocrine-metabolic and body composition benefits for at least up to 12 months after discontinuation. These benefits add to the previously reported beneficial effects on later age at menarche and taller adult height.^1^
